# Real-World Effectiveness of Biologic Therapies for Severe Asthma in a Diverse Middle Eastern Cohort: Evaluation of Longitudinal Symptom Improvement

**DOI:** 10.7759/cureus.105804

**Published:** 2026-03-24

**Authors:** Mohamed Abdul Kareem, Shumaila Z Naqvi, Syed Ammar Husain, Leon Gerard D Cruz, Syed Arshad Husain

**Affiliations:** 1 Department of Pulmonary Medicine, King's College Hospital, Dubai, ARE; 2 Department of Internal Medicine, Maidstone and Tunbridge Wells NHS Trust, Kent, GBR; 3 Department of Research and Innovation, Portsmouth Hospitals University NHS Trust, Portsmouth, GBR; 4 Department of Pulmonary Medicine, Al Zahra Hospital, Dubai, ARE

**Keywords:** a retrospective study, benralizumab, biologics therapy, dupilumab, eosinophil counts, longitudinal monitoring, mepolizumab, real-world effectiveness, severe asthma, tezepelumab

## Abstract

Background and aim

Biologic therapies have transformed the management of severe asthma; however, real-world comparative data from ethnically diverse Middle Eastern populations remain limited. We evaluated the longitudinal effectiveness of benralizumab, dupilumab, mepolizumab, and tezepelumab in routine clinical practice in Dubai. This study aimed to evaluate the real-world performance and effectiveness of biologic therapies for respiratory diseases within the unique, high-dust environment and ethnically diverse patient population of Dubai, thereby providing data to bridge the gap between controlled clinical trials and routine clinical care outcomes.

Methods

This is a retrospective, single-center study of 47 adults with moderate-to-severe asthma receiving biologic therapy, contributing 141 longitudinal observations at baseline, six months, and 12 months. Baseline demographic and biomarker differences were assessed using chi-square and Kruskal-Wallis analyses with Dunn-Bonferroni correction. Longitudinal symptom trajectories (shortness of breath {SOB}, cough, wheeze) were analyzed using generalized estimating equations (GEE) with a binomial distribution and a logit link, accounting for repeated measures. Multivariable logistic regression was used to evaluate predictors of SOB resolution at 12 months.

Results

Significant baseline differences in ethnicity, smoking status, weight, BMI, fractional exhaled nitric oxide (FeNO), and eosinophil counts reflected phenotype-driven biologic selection. Despite this heterogeneity, time on biologic therapy was the only independent predictor of symptom improvement. Each visit was associated with significant reductions in SOB (B = -3.93, p < 0.001), cough (B = -2.28, p < 0.001), and wheeze (B = -1.75, p < 0.001). Biologic class was not associated with differential symptom trajectories. The logistic regression model for SOB resolution demonstrated moderate explanatory power (pseudo R² = 0.397; likelihood ratio test {LRT} p = 0.054), with no biologic showing superiority. Adherence rates were high and adverse events were infrequent.

Discussion

In this real-world, multi-ethnic cohort, biologic therapy was associated with substantial and progressive symptom improvement over 12 months, independent of drug class. These findings support the comparable effectiveness of currently available biologics and reinforce the importance of prolonged treatment durations and longitudinal assessment in the management of severe asthma.

## Introduction

Severe asthma is a specific type of "difficult-to-treat asthma" that stays uncontrolled even after being treated with the highest recommended doses of inhaled corticosteroids (ICS) and long-acting bronchodilators [1]. This long-lasting condition significantly impacts patients' lives, causing them to have poor control over their symptoms, experience frequent flare-ups, and rely more on oral corticosteroids [2]. Regularly taking oral corticosteroids (OCSs) is especially troublesome because it is linked to a wide range of debilitating side effects, such as bone loss, diabetes, and heart problems [3]. This clinical reality creates a pressing and enduring need for effective, steroid-sparing therapies that can reduce the overall disease burden and improve patient quality of life [4]. To improve treatment for severe asthma, there's a critical need for biologic agents targeting key inflammatory mediators. The emergence of biologic therapies has already significantly changed the way we approach this disease [2]. This new approach to treatment emerged because we now have a better understanding of how asthma varies from person to person and of the specific immune processes that cause it [3]. The core pathogenesis involves chronic airway inflammation, which leads to bronchial hyperresponsiveness and variable airflow obstruction. The two dominant endotypes are type 2 (T2)-high asthma, characterized by eosinophilic inflammation driven by cytokines (IL-4, IL-5, IL-13) and epithelial-derived alarmins (thymic stromal lymphopoietin {TSLP}, IL-33), and T2-low asthma, which is often neutrophilic or paucigranulocytic. Recognizing this heterogeneity is essential, as it shifts the therapeutic approach toward precision medicine, where treatment with targeted biologics is matched to a patient's specific underlying endotype [5-7]. Unlike conventional broad-spectrum anti-inflammatory agents, biologic therapies are monoclonal antibodies that precisely modulate the immunoinflammatory cascade by targeting specific cytokines and signaling pathways implicated in the disease [4]. Key classes of these therapies include anti-immunoglobulin E (IgE) (omalizumab), anti-interleukin-5 (IL-5) (mepolizumab), and anti-interleukin-5 receptor alpha (IL-5Rα) (benralizumab), anti-interleukin-4 receptor alpha (IL-4Rα) (dupilumab), and anti-thymic stromal lymphopoietin (anti-TSLP) (tezepelumab) [3]. A significant shift has occurred in the treatment of severe asthma, moving away from a single, one-size-fits-all approach toward a stratified medicine model. With the existence of multiple agents targeting different pathways, studies that provide comparative data are crucial. These studies help clinicians understand how to best apply this personalized approach in clinical practice. The clinical efficacy and safety of biologic therapies have been well-established through rigorous randomized controlled trials (RCTs) [3]. However, a notable research gap remains regarding their performance in "real-world" clinical settings, particularly in patient populations that are ethnically diverse [4]. RCTs are characterized by strict inclusion criteria and highly controlled environments, which can limit the generalizability of their findings to the broader, more heterogeneous patient populations seen in routine practice [8]. Patients in real-world settings often have multiple comorbidities, varying levels of treatment adherence, and a wider range of demographic backgrounds than those included in clinical trials [8]. As such, studies that evaluate these therapies in a real-life context are essential to confirm their effectiveness and safety outside the controlled trial environment [9]. The scarcity of data on severe asthma, not only in the UAE but also across the Middle East, necessitated this study [10].

## Materials and methods

Study setting and design

Patients were selected from the Pulmonology Outpatient Clinic at King's College Hospital Dubai, a leading private healthcare provider in the United Arab Emirates. This retrospective, single-center study evaluated the efficacy of four biologics - benralizumab, dupilumab, mepolizumab, and tezepelumab - in reducing respiratory symptoms in patients with moderate to severe asthma. Symptom data (shortness of breath {SOB}, cough, chest tightness, and wheeze) were collected at baseline, six months, and 12 months. A total of 47 patients contributed 141 longitudinal observations.

Study population

Inclusion and Exclusion Criteria

Adults aged 18-65 years with moderate-to-severe asthma, followed in the pulmonology clinic by a senior pulmonologist, and eligible for biologic therapy were included in this analysis. Patients with mild asthma, age <18 or >65 years, or a diagnosis of conditions other than asthma (e.g., chronic obstructive pulmonary disease {COPD} or asthma-COPD overlap syndrome) were excluded from this study.

Data collection

Data were retrospectively extracted from the electronic medical record (EMR) system (Cerner), including demographics (age, sex, and ethnicity); clinical history (allergies, comorbidities, and prior asthma control); baseline diagnostics (spirometry {forced expiratory volume in 1 second (FEV₁), forced vital capacity (FVC), maximal expiratory flow at 25% of the forced vital capacity (MEF25)}, FeNO, IgE, eosinophil counts; symptom data (SOB, cough, wheeze {recorded from physician notes at each time point}). The symptoms were coded as binary outcomes. All data were anonymized and stored securely in password-protected files.

Statistical analysis

Analyses were conducted to describe demographic and clinical characteristics, evaluate treatment effects, and assess longitudinal symptom trajectories. Adherence was operationally defined as a binary categorical variable (compliant vs. non-compliant). A patient was categorized as "compliant" if the physician’s clinical documentation confirmed consistent administration of the prescribed therapy. This binary measure was applied consistently to evaluate proportional differences across biologic groups using weighted binomial tests and as a predictor in the multivariable logistic regression model.

Baseline Comparisons

Categorical variables (sex, smoking status, ethnicity, pet ownership) were compared across biological groups using χ² tests. Monte Carlo simulation (10,000 resamples, 99% confidence level) was applied for sparse contingency tables. Continuous variables (weight, BMI, FeNO, eosinophil count) were compared using Kruskal-Wallis tests, with significant omnibus results followed by Dunn-Bonferroni-corrected post hoc comparisons.

Longitudinal Symptom Analysis

Symptom trajectories for SOB, cough, and wheeze were evaluated using generalized estimating equations (GEE) with a binomial distribution and logit link function, accounting for repeated measures with an exchangeable correlation structure. Time on biologic therapy (visits 1, 2, and 3) was included as a continuous predictor, alongside covariates such as biologic type, sex, age, BMI, FeNO, eosinophil counts, and smoking status. Model effects were assessed using Wald chi-square tests, with statistical significance set at p < 0.05. Regression coefficients (B), standard errors (SE), and 95% confidence intervals (CI) were reported.

Predictors of SOB Resolution

Given that SOB demonstrated the most pronounced longitudinal improvement, multivariable logistic regression (MLR) was used to evaluate predictors of SOB resolution at 12 months. Covariates included biologic type, baseline FeNO, baseline eosinophil count, and compliance with biologic therapy. Model performance was assessed using pseudo R² and likelihood ratio tests. Odds ratios (ORs), 95% CIs, and p-values were reported.

## Results

Demographic analysis

A total of 47 patients with severe asthma contributed longitudinal data, yielding 141 observations across three time points (baseline, six months, and 12 months) (Tables 1, 2). Baseline demographic characteristics were compared across patients initiating benralizumab, dupilumab, mepolizumab, or tezepelumab. There was no statistically significant difference in sex distribution across treatment groups (χ² test, p = 0.607), and pet ownership also did not differ significantly (χ² test, p = 0.564; Monte Carlo, p = 0.585). In contrast, nationality demonstrated a highly significant association with biologic allocation (χ² test, p < 0.001; Monte Carlo, p < 0.001), as did recoded ethnicity (χ² test, p < 0.001; Monte Carlo, p < 0.001). Smoking status also differed significantly across groups (χ² test, p < 0.001; Monte Carlo, p < 0.001). Monte Carlo simulation with 10,000 resamples, and a 99% confidence level was applied where contingency tables contained sparse cells or zero frequencies, thereby ensuring robust estimation of p-values.

**Table 1:** Comparison of baseline characteristics and biologic treatment effects across different biologic groups. *P < 0.05 was statistically significant. **P < 0.01 was statistically significant. ***P < 0.001 was statistically significant. Presented here is a comparative analysis of baseline characteristics (age, height, weight, BMI, SpO_2_) and treatment outcomes (FeNO and eosinophils at baseline and after six months of biologic treatment) across the following four different biologic groups: benralizumab, dupilumab, mepolizumab, and tezepelumab. Mean values and standard deviation (SD) within each biologic group are provided for each variable, alongside p-values calculated from Kruskal-Wallis analysis, indicating the statistical significance of differences between the groups. Where statistical significance is indicated, post-hoc Dunn-Bonferroni tests were conducted and described in the main text. FeNO: fractional exhaled nitric oxide

Patients (n = 47)	Biologic								p-Value	Significance
	Benralizumab (n = 17)		Dupilumab (n = 15)		Mepolizumab (n = 7)		Tezepelumab (n = 8)			
	Mean	SD	Mean	SD	Mean	SD	Mean	SD		
Age (years)	45	4	45	14	40	8	46	3	0.167	ns
Height (cm)	166.3	8.4	168.2	8.6	168.4	6.3	168.9	10.3	0.558	ns
Weight (kg)	68.49	13.25	80.73	15.01	71.81	12.40	74.63	16.04	0.002	**
BMI (kg/m^2^)	25	5	28	5	25	5	26	4	0.006	**
SpO_2_ (%)	97	1	98	1	98	1	98	1	0.051	ns
FeNO at start of study (parts per billion)	75	50	65	58	58	36	25	19	<0.001	***
FeNO after commencement of biologics at 6 months (parts per billion)	22	32	17	14	22	21	7	7	0.038	*
Eosinophils at start of study (10^9^/L)	1.009	1.315	0.895	0.879	0.435	0.341	0.138	0.049	<0.001	***
Eosinophils after commencement of biologics at 6 months (10^9^/L)	0.264	0.502	0.482	0.373	0.419	0.479	0.178	0.130	<0.001	***

**Table 2:** Baseline demographic and exposure characteristics of patients stratified by biologic therapy. Presented here are the distributions of baseline demographic characteristics (sex, nationality, and ethnicity), smoking status, and pet ownership across patients treated with benralizumab, dupilumab, mepolizumab, and tezepelumab. Data are expressed as counts and column percentages. Group differences were assessed using omnibus chi-square tests, with Monte Carlo simulation (10,000 iterations; 99% confidence level) applied where appropriate due to sparse cell counts.

Variables		Biologic								p-Value	p-Value (Monte Carlo)
		Benralizumab		Dupilumab		Mepolizumab		Tezepelumab			
		n	%	n	%	n	%	n	%		
Sex	Male	24	0.471	18	0.400	12	0.571	12	0.500	0.607	0.608
	Female	27	0.529	27	0.600	9	0.429	12	0.500		
Nationality	Bangladesh	3	0.059	0	0.000	0	0.000	0	0.000		
	British	9	0.176	9	0.200	0	0.000	12	0.500		
	India	24	0.471	6	0.133	6	0.286	3	0.125		
	Japanese	0	0.000	0	0.000	0	0.000	3	0.125		
	Jordanian	3	0.059	3	0.067	0	0.000	0	0.000		
	Morocco	3	0.059	0	0.000	0	0.000	0	0.000		
	Netherlands	0	0.000	0	0.000	0	0.000	3	0.125	<0.001	<0.001
	Pakistan	3	0.059	12	0.267	12	0.571	3	0.125		
	Saudi Arabia	0	0.000	3	0.067	0	0.000	0	0.000		
	South Africa	0	0.000	3	0.067	0	0.000	0	0.000		
	Sri Lanka	0	0.000	3	0.067	0	0.000	0	0.000		
	Sudan	0	0.000	0	0.000	3	0.143	0	0.000		
	UAE	6	0.118	6	0.133	0	0.000	0	0.000		
Ethnicity	African	0	0.000	0	0.000	3	0.143	0	0.000		
	East Asian	0	0.000	0	0.000	0	0.000	3	0.125		
	Middle Eastern	15	0.294	12	0.267	0	0.000	0	0.000	<0.001	<0.001
	South Asian	36	0.706	24	0.533	18	0.857	12	0.500		
	White	0	0.000	9	0.200	0	0.000	9	0.375		
Smoking status	Ex-smoker	0	0.000	3	0.067	0	0.000	3	0.125		
	No	45	0.882	42	0.933	18	0.857	18	0.750	<0.001	<0.001
	Shisha	6	0.118	0	0.000	0	0.000	3	0.125		
	Yes	0	0.000	0	0.000	3	0.143	0	0.000		
Pet ownership	Have pets	48	0.941	42	0.933	18	0.857	21	0.875	0.564	0.585
	No pets	3	0.059	3	0.067	3	0.143	3	0.125		

Assessment of differences in clinical variables across biologic treatment groups

Kruskal-Wallis (KW) analyses were conducted to assess differences in scalar clinical variables between biologic groups. Significant omnibus KW tests were followed by Dunn-Bonferroni corrected pairwise comparisons. Significant between-group differences were observed for weight (omnibus p < 0.001), with post hoc testing demonstrating a significant difference between benralizumab and dupilumab (adjusted p < 0.001). BMI also differed significantly across treatment arms (omnibus p = 0.006), with a significant pairwise difference between benralizumab and dupilumab (adjusted p = 0.004).

Baseline fractional exhaled nitric oxide (FeNO) levels differed significantly across biologic groups (omnibus p < 0.001). Dunn-Bonferroni post hoc testing demonstrated that patients commencing tezepelumab had significantly different baseline FeNO compared with dupilumab (adjusted p = 0.004), mepolizumab (adjusted p = 0.006), and benralizumab (adjusted p < 0.001).

Following biologic initiation, FeNO remained significantly different at the omnibus level (p = 0.038). However, pairwise differences did not retain statistical significance after Bonferroni correction (tezepelumab vs. dupilumab adjusted p = 0.052; tezepelumab vs. mepolizumab adjusted p = 0.102).

Significant differences in baseline blood eosinophil absolute count (EAA) were observed (omnibus p < 0.001). Patients initiating tezepelumab demonstrated significantly different baseline EAA compared with mepolizumab (adjusted p = 0.021), dupilumab (adjusted p < 0.001), and benralizumab (adjusted p < 0.001). After biologic initiation, significant differences persisted between benralizumab and dupilumab (adjusted p < 0.001), with a borderline difference between tezepelumab and dupilumab (adjusted p = 0.050). All other pairwise comparisons were non-significant following correction.

Binomial analyses evaluating differences in treatment, adverse events, and compliance

Weighted binomial test analyses were conducted to evaluate proportional differences in treatment utilization, adverse events, and compliance variables, with cases weighted by yes/no frequency responses. A significantly greater proportion of patients remained compliant with inhaled corticosteroids (ICS) therapy following biologic initiation (p < 0.01). Adverse events were infrequent, with 12 recorded events compared to 129 non-events (p < 0.01). Anti-leukotriene therapy was significantly prevalent prior to biologic initiation (p < 0.01) and remained significantly maintained during follow-up (p < 0.01), although discontinuation was more frequently observed in the mepolizumab arm. Compliance with biologic therapy itself was significantly higher than non-compliance (p < 0.01). Similarly, montelukast adherence was significantly greater than non-adherence across biologic groups (p < 0.01), with the exception of mepolizumab, where proportional differences were less marked. ICS inhaler compliance was also significantly higher than non-compliance overall (p < 0.01).

Longitudinal symptom outcome evaluation with generalized estimating equation (GEE) models

Generalized estimating equation (GEE) models were built with a binomial distribution and logit link function, accounting for repeated within-patient observations using an exchangeable working correlation structure (Tables 3, 4). Estimated working correlations were small (approximately -0.02 to 0.04), indicating minimal residual within-subject correlation after covariate adjustment.

**Table 3:** GEE models examining longitudinal predictors of respiratory symptoms (SOB, cough, and wheeze) under the treatment effects of four different biologics. Reports parameter estimates (B), standard errors (SE), 95% confidence intervals (CI), and p-values for statistically significant predictors. SOB: shortness of breath; GEE: generalized estimating equation

Symptom	Significant predictor	Parameter estimate (B)	SE	95% CI	p-Value
SOB	Visit number	-3.93	0.99	-5.87 to -1.99	<0.001
Cough	Visit number	-2.28	0.59	-3.44 to -1.12	<0.001
Wheeze	Visit number	-1.75	0.39	-2.51 to -1.00	<0.001

**Table 4:** GEE models examining longitudinal predictors of respiratory symptoms (SOB, cough, and wheeze) under the treatment effects of four different biologics. Displays Wald χ² statistics and corresponding p-values for all predictors included in the models. Across all three symptom models, visit number was the only statistically significant predictor. Increasing visit number was associated with significant reductions in symptom scores for SOB (B = -3.93; SE = 0.99; 95% CI: -5.87 to -1.99; p < 0.001), cough (B = -2.28; SE = 0.59; 95% CI: -3.44 to -1.12; p < 0.001), and wheeze (B = -1.75; SE = 0.39; 95% CI: -2.51 to -1.00; p < 0.001). Wald χ² statistics confirmed the robustness of this effect (all p < 0.001). No significant associations were observed for age, BMI, weight, height, or oxygen saturation (SpO₂) in any of the models (all p > 0.05). Intercepts were also non-significant. The confidence intervals for visit number did not cross zero in any model, indicating consistent and statistically reliable decreases in symptom burden over time. SOB: shortness of breath; GEE: generalized estimating equation

Predictor	SOB Wald χ² (p-Value)	Cough Wald χ² (p-Value)	Wheeze Wald χ² (p-Value)
Intercept	0.015 (0.903)	0.208 (0.648)	3.452 (0.063)
Visit number	15.773 (<0.001)	14.816 (<0.001)	20.593 (<0.001)
Age (years)	0.693 (0.405)	0.842 (0.359)	0.347 (0.556)
BMI (kg/m^2^)	0.237 (0.626)	0.446 (0.504)	1.188 (0.276)
Weight (kg)	0.476 (0.490)	0.028 (0.866)	0.952 (0.329)
Height (cm)	0.962 (0.327)	0.984 (0.321)	2.657 (0.103)
SpO₂ (%)	3.098 (0.078)	0.057 (0.811)	0.446 (0.504)

Time on biologic therapy was a consistent and statistically significant predictor of symptom reduction across all domains (Tables 3, 4). For shortness of breath (SOB), each incremental visit was associated with a significant reduction in the odds of symptom presence (B = -3.93; SE = 0.99; Wald χ² = 15.77; p < 0.001; 95% CI -5.87 to -1.99). For cough, visit number remained the sole significant predictor (B = -2.28; SE = 0.59; Wald χ² = 14.82; p < 0.001; 95% CI -3.44 to -1.12). Similarly, wheeze decreased significantly over time (B = -1.75, SE = 0.39; Wald χ² = 20.59; p < 0.001; 95% CI -2.51 to -1.00). Biologic class was not independently associated with differential symptom trajectories in either simplified or fully adjusted models (p = 0.12-0.31).

The magnitude of the effect was greatest for SOB (B = -3.93), followed by cough (B = -2.28) and wheeze (B = -1.75), suggesting that dyspnea showed the most pronounced longitudinal improvement. The negative regression coefficients indicate a downward trajectory in symptom severity with increasing follow-up, and the narrow confidence intervals, none crossing zero, support the stability of these findings. Overall, the findings indicate that symptom burden declined significantly over time, independent of measured covariates, highlighting a robust longitudinal improvement (longer time on biologics, better outcomes) pattern across respiratory outcomes.

Multivariable logistic regression (MLR) analysis to determine predictors of SOB resolution at 12 months

As SOB was shown to be the symptom with the most improvement longitudinally, MLR models were constructed to evaluate the predictors of SOB resolution at 12 months. Metrics of the model are shown in Table 5. The MLR model included the following variables: biologic type, baseline FeNO, baseline eosinophil count, and compliance with biologic therapy (Table 6). The overall model demonstrated moderate explanatory power (pseudo R² = 0.397) and approached statistical significance (likelihood ratio test p = 0.054) (Table 5). After adjustment, biologic type was not independently associated with symptom resolution. Compared with benralizumab, neither dupilumab (p = 0.791), mepolizumab (p = 0.903), nor tezepelumab (p = 0.619) demonstrated significantly different odds of improvement (Table 6).

**Table 5:** MLR model examining predictors of improvement in SOB. MLR was scripted using Python’s statsmodels library. Results shown here from an MLR analysis evaluating factors associated with improvement in shortness of breath (improved_sob; binary outcome). Model characteristics are shown in panel (a), including the number of observations, model degrees of freedom, log-likelihood statistics, pseudo R², and likelihood ratio (LR) test results. Panel (b) displays regression coefficients (log-odds), standard errors (SE), z-statistics, p-values, and 95% confidence intervals (CI) for each predictor. Unlike linear R², pseudo R² values are not directly interpretable as proportion of variance explained, but this value indicates moderate model fit. The model log-likelihood (-9.40) improved compared with the null model (-15.58), meaning the predictors collectively improved model fit. The LRT p-value indicates whether the full model fits significantly better than a model with no predictors. The p-value 0.054 indicates borderline statistical significance. Covariance type: non-robust means that standard errors were calculated using the conventional maximum likelihood (model-based) method, while not using heteroskedasticity-robust “sandwich” corrections. In small samples (n = 44), non-robust standard errors are common and acceptable as heteroskedasticity was not a concern here. The analytic sample excludes three patients from the original cohort (n = 47) who did not report shortness of breath (SOB) at baseline, as improvement could only be assessed for symptomatic individuals. MLR: multivariable logistic regression; SOB: shortness of breath; MLE: maximum likelihood estimation; LL-null: log-likelihood of the null model; LRT: likelihood ratio test

Parameters	Values
Dependent variable	Improved shortness of breath (improved_SOB)
Number of observations	44
Model type	Logistic regression (Logit)
Method	MLE
Degrees of freedom (model)	6
Degrees of freedom (residuals)	37
Log-likelihood	-9.3978
LL-Null	-15.578
Pseudo R²	0.3967
LRT p-value	0.05439
Model convergence	True
Covariance type	Nonrobust (standard MLE-based standard errors)

**Table 6:** MLR model examining predictors of improvement in SOB. Biologic therapy was entered as a categorical variable with benralizumab as the reference category; coefficients for dupilumab, mepolizumab, and tezepelumab therefore represent effects relative to benralizumab. Continuous predictors included fractional exhaled nitric oxide (FeNO) at presentation and eosinophil count. Treatment compliance was entered as a binary variable. Covariance type was specified as non-robust (model-based standard errors). MLR: multivariable logistic regression; SOB: shortness of breath

Variables	Coefficient	Standard error	Z	p>|z|	95% CI (0.025-0.975)
Intercept	-8.8388	6.932	-1.275	0.202	-22.425 to 4.747
Effect of dupilumab vs. benralizumab	0.4187	1.579	0.265	0.791	-2.676 to 3.513
Effect of mepolizumab vs. benralizumab	8.8974	72.927	0.122	0.903	-134.037 to 151.832
Effect of tezepelumab vs. benralizumab	0.8884	1.788	0.497	0.619	-2.616 to 4.393
FeNO at presentation (parts per billion)	0.0569	0.037	1.53	0.126	-0.016 to 0.13
Eosinophils (x10^9^/L)	0.4325	0.779	0.555	0.579	-1.094 to 1.959
Compliance on biologics	8.6849	5.659	1.535	0.125	-2.406 to 19.776

**Figure 1:**
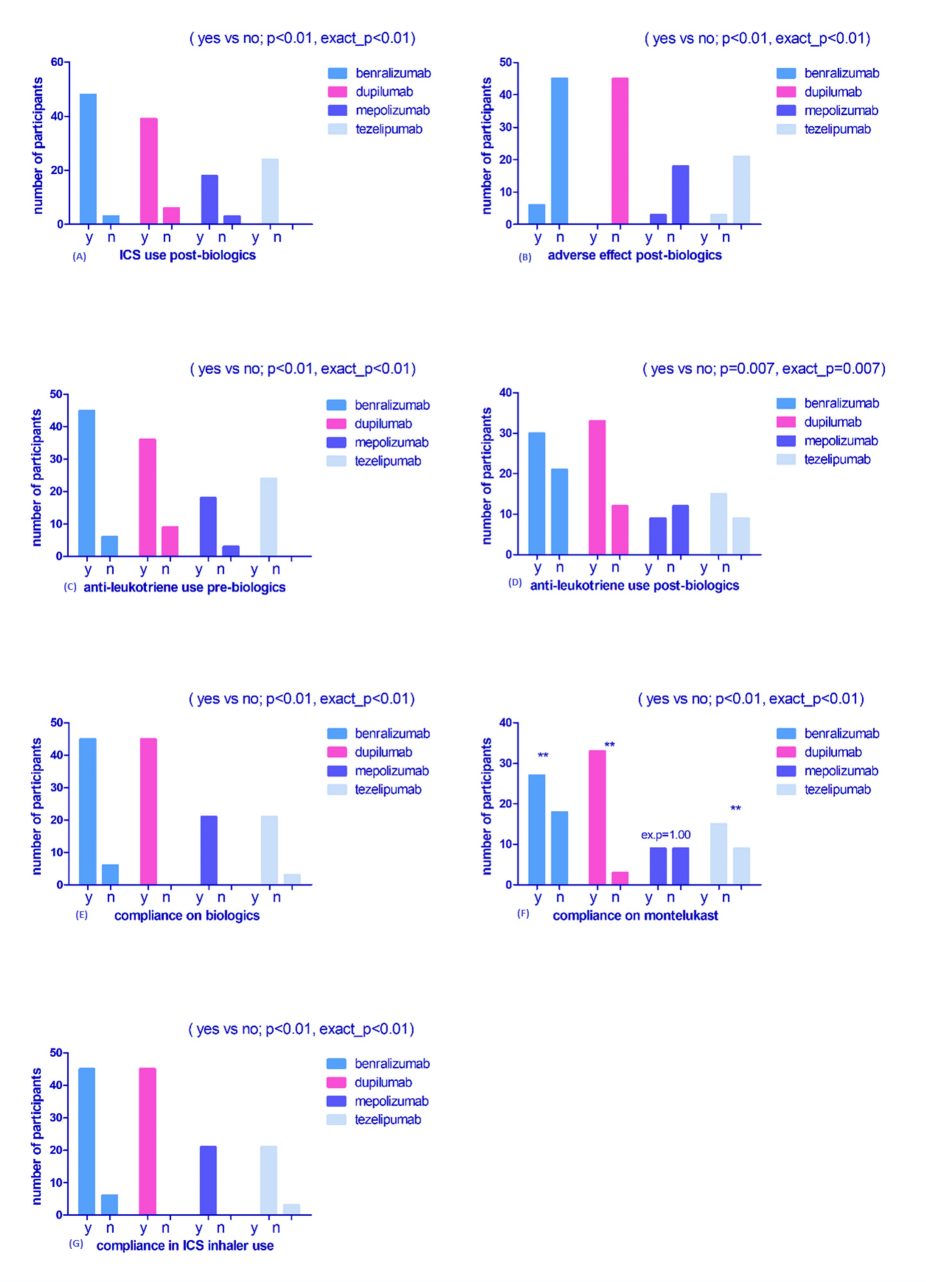
Distribution of treatment patterns, adverse effects, and medication compliance across four biologic therapies. Weighted binomial test analyses (SPSS; Armonk, NY: IBM Corp.) were performed for each variable, with cases weighted by frequency of yes/no responses. Exact p-values are displayed on each subplot. Graphs illustrate frequency distributions across the four biologics. (A) Inhaled corticosteroid (ICS) use post-biologic initiation bar chart showing weighted frequencies of continued inhaled corticosteroid (ICS) use after biologic commencement across the four biologics. (B) Adverse effects due to biologics distribution of reported biologic-related adverse effects (yes/no) by biologic type. (C) Anti-leukotriene use pre-biologic frequency of anti-leukotriene therapy use prior to biologic initiation across treatment groups. (D) Anti-leukotriene use post-biologic weighted frequencies of continued anti-leukotriene therapy following biologic commencement. (E) Compliance with biologics while on treatment, distribution of biologic adherence (compliant vs. non-compliant) during treatment. (F) Compliance with montelukast while on biologic therapy weighted adherence rates to montelukast among patients receiving biologic therapy. (G) Compliance with ICS inhaler while on biologic therapy, and distribution of ICS inhaler adherence during biologic treatment.

Higher baseline FeNO levels showed a positive but non-significant association with symptom resolution (OR: 1.06 per unit increase, p = 0.126). Baseline eosinophil count was not significantly associated with improvement (p = 0.579). Compliance with biologic therapy demonstrated a strong positive effect size, although this did not reach statistical significance (p = 0.125) (Figure 1).

## Discussion

This real-world longitudinal analysis examined demographic heterogeneity, inflammatory biomarkers, medication utilization patterns, adherence, and symptom trajectories among patients receiving benralizumab, dupilumab, mepolizumab, or tezepelumab for severe asthma. Significant baseline differences were observed in demographic measures and inflammatory biomarkers, reflecting phenotype-driven prescribing practices. Despite this heterogeneity, longitudinal modeling demonstrated that time on biologic therapy was the dominant determinant of clinical improvement, with substantial and progressive reductions in SOB, cough, and wheeze across all biologic classes. No biologic demonstrated superiority in symptom trajectory over 12 months. However, given the modest sample size, these comparative results are exploratory.

Baseline differences in FeNO and eosinophil counts were consistent with mechanism-based selection of therapy. Persistent post-treatment differences in eosinophil counts, particularly between benralizumab and dupilumab, likely reflected pharmacodynamic variation rather than differential clinical effectiveness. Importantly, attenuation of FeNO differences after treatment suggested partial convergence of inflammatory activity across biologic classes.

High levels of biologic and ICS adherence, together with a low adverse event rate, supported favorable tolerability in routine practice. Variability in montelukast continuation may reflect clinician-directed step-down decisions or differences in perceived adjunct benefit.

The use of GEE modeling was particularly important in this study. Standard cross-sectional statistical tests, such as the Kruskal-Wallis test or chi-square test, assess between-group differences at a single time point but do not account for repeated measures within individuals. In longitudinal datasets, repeated observations from the same patient could be inherently correlated, violating the independence assumption of conventional regression models. GEE addresses this by modeling population-averaged effects while explicitly accounting for intra-individual correlation through a working correlation structure. This approach allowed estimation of the independent effect of time on symptom improvement while adjusting for demographic, anthropometric, and treatment-related covariates.

Importantly, GEE provided robust standard errors even when the correlation structure was modest, thereby ensuring valid inference. Thus, the analysis demonstrated that symptom improvement was a consistent time-dependent effect rather than a function of biologic class or baseline differences. GEE enabled identification of the principal clinical signal in this study as follows: sustained symptomatic improvement over time across heterogeneous biologic therapies.

In this adjusted analysis, biologic therapy was associated with high rates of shortness of breath resolution across all treatment groups. After accounting for baseline inflammatory markers and treatment adherence, no individual biologic agent demonstrated superiority over others.

The absence of statistically significant differences between biologics suggested comparable effectiveness within this cohort. Notably, mepolizumab exhibited uniformly favorable outcomes, which contributed to statistical quasi-separation in the regression model. This phenomenon reflected the very high response rate observed rather than model misspecification.

Baseline FeNO demonstrated a positive trend toward predicting response, suggesting that type 2 inflammatory burden may influence clinical improvement. However, this association did not reach statistical significance, likely reflecting limited statistical power. Similarly, baseline eosinophil count was not independently associated with symptom resolution after adjustment.

Compliance with biologic therapy showed a strong effect size, supporting the clinical importance of adherence in achieving optimal outcomes. Although this finding did not reach statistical significance, the magnitude of the coefficient suggested that adherence may meaningfully influence treatment response in larger cohorts.

Overall, the findings indicated that symptom resolution was common across biologic therapies, and no clear evidence of differential effectiveness was observed after adjustment for baseline inflammatory markers and adherence. The high response rate and relatively small subgroup sizes limited the precision of effect estimates. Larger prospective studies may help clarify whether subtle differences exist between agents or whether treatment response is primarily phenotype-driven rather than drug-specific. Collectively, these findings support the real-world effectiveness of biologic therapy in severe asthma across diverse patient populations and reinforce the importance of longitudinal evaluation when assessing treatment response.

Limitations and future research

The primary limitations of this study include the modest sample size (47 patients), which limits statistical power and the ability to generalize the findings, and the reliance on self-reported binary symptom outcomes, which may underestimate nuanced changes in severity. Crucially, the single-center bias limits the external validity of the results, especially for a broader severe asthma population, and the lack of objective lung function outcomes (FEV₁) prevents a complete assessment of treatment efficacy by correlating symptomatic improvement with physiological changes. Furthermore, the real-world setting introduced unmeasured potential confounders, such as variability in patient adherence to standard-of-care medications and uncontrolled environmental dust exposure, all of which could independently influence symptom reporting and obscure the true differential effects of the biologic therapies.

## Conclusions

In conclusion, this real-world study demonstrates that biologic therapies, specifically benralizumab, dupilumab, mepolizumab, and tezepelumab, are highly effective in reducing the burden of severe asthma within a diverse, multi-ethnic Middle Eastern population. Despite significant baseline differences in patient demographics and inflammatory biomarkers that initially guided the selection of specific drugs, the analysis revealed that time on therapy was the primary independent predictor of clinical success. Over a 12-month period, patients experienced substantial and progressive improvements in key symptoms, including shortness of breath, cough, and wheeze, with no single biologic demonstrating clinical superiority over the others. Supported by high adherence rates and a low incidence of adverse events, these findings affirm the comparable effectiveness of currently available biologics in routine practice and underscore the necessity of sustained, longitudinal assessment to achieve optimal therapeutic outcomes in severe asthma management.
